# Increased Alzheimer's risk during the menopause transition: A 3-year longitudinal brain imaging study

**DOI:** 10.1371/journal.pone.0207885

**Published:** 2018-12-12

**Authors:** Lisa Mosconi, Aneela Rahman, Ivan Diaz, Xian Wu, Olivia Scheyer, Hollie Webb Hristov, Shankar Vallabhajosula, Richard S. Isaacson, Mony J. de Leon, Roberta Diaz Brinton

**Affiliations:** 1 Department of Neurology, Weill Cornell Medical College, New York, NY, United States of America; 2 Department of Psychiatry, New York University School of Medicine, New York, NY, United States of America; 3 Division of Biostatistics and Epidemiology, Department of Healthcare Policy and Research, Weill Cornell Medicine, New York, NY, United States of America; 4 Department of Radiology, Weill Cornell Medical College, New York NY, United States of America; 5 Departments of Pharmacology and Neurology, College of Medicine, University of Arizona, Tucson, United States of America; Nathan S Kline Institute, UNITED STATES

## Abstract

Two thirds of all persons with late-onset Alzheimer’s disease (AD) are women. Identification of sex-based molecular mechanisms underpinning the female-based prevalence of AD would advance development of therapeutic targets during the prodromal AD phase when prevention or delay in progression is most likely to be effective. This 3-year brain imaging study examines the impact of the menopausal transition on Alzheimer’s disease (AD) biomarker changes [brain β-amyloid load via ^11^C-PiB PET, and neurodegeneration via ^18^F-FDG PET and structural MRI] and cognitive performance in midlife. Fifty-nine 40–60 year-old cognitively normal participants with clinical, neuropsychological, and brain imaging exams at least 2 years apart were examined. These included 41 women [15 premenopausal controls (PRE), 14 perimenopausal (PERI), and 12 postmenopausal women (MENO)] and 18 men. We used targeted minimum loss-based estimation to evaluate AD biomarker and cognitive changes. Older age was associated with baseline Aβ and neurodegeneration markers, but not with rates of change in these biomarkers. APOE4 status influenced change in Aβ load, but not neurodegenerative changes. Longitudinally, MENO and PERI groups showed declines in estrogen-dependent memory tests as compared to men (p < .04). Adjusting for age, APOE4 status, and vascular risk confounds, the MENO and PERI groups exhibited higher rates of CMRglc decline as compared to males (p ≤ .015). The MENO group exhibited the highest rate of hippocampal volume loss (p’s ≤ .001), and higher rates of Aβ deposition than males (p < .01). CMRglc decline exceeded Aβ and atrophy changes in all female groups vs. men. These findings indicate emergence and progression of a female-specific hypometabolic AD-endophenotype during the menopausal transition. These findings suggest that the optimal window of opportunity for therapeutic intervention to prevent or delay progression of AD endophenotype in women is early in the endocrine aging process.

## Introduction

Female sex is a major risk factor for developing late-onset Alzheimer’s disease (AD)[1]. While AD is not unique to females, women constitute two-thirds of patients living with AD-dementia worldwide[2]. These findings are largely independent of women’s greater longevity relative to men[3].

The biological mechanisms underlying the increased AD risk in women are not fully understood. Preclinical evidence has implicated a shift in the glucose bioenergetic system of the brain during the *perimenopause to menopause transition* (PTMT) as an early initiating mechanism[4]. The PTMT is a midlife neuroendocrine transition state that culminates with reproductive senescence. However, symptoms are largely neurological in nature, including disruption of estrogen-regulated systems such as thermoregulation, sleep, and circadian rhythms, as well as depression and cognitive decline[4].

Estrogen is a well-known “master regulator” of the metabolic system of the female brain and body. Within the brain, estrogen regulates glucose transport, aerobic glycolysis, and mitochondrial function to generate ATP in multiple brain regions involved in cognitive functions, such as medial temporal, posterior cingulate and frontal cortex [5].

However, the cognitive effects of estrogen declines have been difficult to pinpoint by means of standard cognitive testing. It is well-documented that women perform significantly better than age-matched men across several cognitive measures, especially verbal memory tests[6,7], which may hinder detection of early AD-related changes in women [8]. In fact, some studies suggest this advantage persist into early AD, though others indicate an attenuation after menopause [9].There is emerging evidence that some cognitive tests are more sensitive to declines in memory and higher-order processing related to decreasing brain estrogen levels in women[10]^,^[11], [12] although these observations have not been validated in terms of future AD risk or with the use of biomarkers.

Neuroimaging studies have provided clearer evidence that the menopause transition increases risk of AD-related brain changes in women. Our previous cross-sectional studies have shown that perimenopausal and postmenopausal women exhibit an AD-endophenotype characterized by increased brain amyloid-beta (Aβ) deposition (a hallmark of AD pathology), hypometabolism, and neuronal volume loss compared to premenopausal women and men of similar age[13],[14]. Across all modalities, the frontal cortex and posterior cingulate cortex showed consistent biomarker abnormalities in peri- and postmenopausal women[13],[14]. Additionally, women of menopausal age showed reduced gray matter volumes in parietal and temporal regions, including medial temporal cortex, as compared to men[13]. It remains to be established whether these biomarker abnormalities are progressive, and therefore indicative of an ongoing AD process.

Herein, we report 3-year follow-up brain imaging observations of the previously studied cohorts. This observational longitudinal study characterizes progression of well-established AD-biomarkers [^11^C-PiB PET Aβ deposition, and neurodegeneration via ^18^F-FDG PET glucose metabolism and MRI atrophy], in cognitively normal women at different endocrine transition stages (pre-menopause, perimenopause, menopause), and men, adjusted by age and other potential confounders.

## Methods

### Participants

Study participants were derived from a longitudinal brain imaging study of risk factors for AD among clinically and cognitively normal adults, conducted at New York University (NYU) School of Medicine /Weill Cornell Medicine (WCM) between 2010–2016. Details about the study design have previously been published[13],[14].

Only those with clinical, laboratory, neuropsychological, and brain imaging including MRI, FDG- and PiB-PET at least 2 years apart (range 2–3.5 years) were examined. Participants had to be 40–60 years old at baseline, with education≥12 years, Clinical Dementia Rating = 0, Global Deterioration Scale≤2, Mini Mental State Examination≥27, Hamilton depression scale<16, and normal cognitive test performance for age and education[13],[14].

Those with medical conditions or history of conditions that may affect brain structure or function (i.e. stroke, diabetes, head trauma, neurodegenerative diseases, depression, hydrocephalus, intracranial mass and infarcts on MRI), and those taking psychoactive medications were excluded. Women with thyroid disease or full hysterectomy, and those on hormonal replacement therapy (HRT) were also excluded.

A family history of AD that included at least one 1^st^ degree relative whose AD onset was after age 60 was elicited using standardized questionnaires[15]. Apolipoprotein E (APOE) genotypes were determined using standard qPCR procedures[15].

### Standard protocol approvals, registrations, and patient consents

Written informed consent was obtained from all subjects in this NYU/WCM institutional review board-approved study.

### Menopausal status

Determination of menopausal status was based on clinical judgment, medical records, and detection of cluster symptoms according to the Stages of Reproductive Aging Workshop (STRAW) criteria[16], as previously described[13],[14]. Cluster symptoms included presence of sweatiness, hot flashes, mood swings, insomnia, appetite changes, loss of libido, cognitive problems, poor concentration, and short-term memory complaints. Based on these assessments, female participants were classified as premenopausal controls (PRE); perimenopausal (PERI); and postmenopausal (MENO).

### Cognitive measures

The neuropsychological battery was previously described [13],[14]. Three cognitive domains were assessed at both time points: memory (immediate and delayed recall of a paragraph and paired associates), higher-order processing (Wechsler Adult Intelligence Scale [WAIS] digit symbol and block design tests), and language (WAIS vocabulary and object naming). These measures were used to compute the global cognitive summary score by z-scoring each measure within each domain, and then averaging each component of the three domains, at each time point. The majority of brain imaging studies investigating AD risk factors in midlife focused on global cognitive summary scores as the main cognitive outcome variable (for a review of findings see [17]). In order to compare our data to previous publications, we used global cognition as the main cognitive outcome. Secondly, based on previous observations that specific tests are selectively sensitive to estrogen changes, and could therefore better capture early cognitive decline in middle-aged women, we also examined changes in estrogen-dependent memory (paragraph recall) and higher-order processing (block design) tests as female-specific outcomes[10,14]^,^[11].

### Brain imaging

All subjects received MRI, PiB- and FDG-PET scans at baseline and at least 2 years later following standardized protocols[13],[14].

3D volumetric T1 magnetization-prepared rapid gradient echo (MPRAGE) scans were obtained on 3T MRI scanners. Freesurfer software (version 5.3) with a longitudinal processing pipeline was used to obtain total hippocampal volumes on longitudinal MRI scans[18]. Total intracranial volumes (TIV) were also estimated.

PET images were acquired with PET/CT scanners operating in 3D mode. All PET quantitative image analyses were performed using a fully automated image processing pipeline. Statistics on image voxel values were extracted from automatically labeled cortical regions of interest using the automated anatomic labeling atlas (AAL)[19]. We selected the posterior cingulate cortex (PCC) as the target AD-related region-of-interest (ROI), and the prefrontal cortex as the target aging-related ROI[20]. Standardized uptake value ratios (SUVR) were generated by normalizing ROI PiB uptake by cerebellar gray matter ROI of the atlas, and ROI FDG uptake by global activity [13],[14].

### Statistics

R v.3.4.1 and SPSS v.23 were used for data analysis. Clinical and demographic measures were examined with χ^2^ tests and general linear models, at p<0.05.

For biomarker and cognitive measures, we used targeted minimum loss-based estimation (TMLE)[21] to compute adjusted means for each group (PRE, PERI, MENO, and men). Our estimates thus reflect the difference in outcomes between the groups after all possible measured confounding has been removed. TMLE uses a preliminary estimator of the outcome regression and propensity score to construct a doubly robust estimator that remains consistent under misspecification of either model[21]. The estimator of the conditional distribution for the measures, as well as the model for predicting group membership as a function of covariates, i.e. the “propensity score”, were estimated using a cross-validation selector that chooses the best fitting model among the following candidate models [22]: stepwise regression, elastic net generalized linear models, multivariate adaptive splines, and generalized additive models. More details about the TMLE methodology are included in Appendix.

Age, education, and APOE4 status (carriers vs. non-carriers) were examined as potential confounders. While none of our participants had overt cardio- and/or cerebro-vascular disease, there is evidence that vascular risk factors influence brain aging even in those without a diagnosis of these conditions [23]. As such, overweight (e.g., BMI>25 kg/m^2^), hypertension, and insulin resistance by means of Quantitative Insulin Sensitivity Check Index, QUICKI scores[24] were further examined as possible confounders. MRI analyses were adjusted for TIV. PET SUVRs are normalized by reference values.

Results were considered significant at p < .05 corrected for multiple comparisons (e.g., outcomes), yielding a conservative p-value that controls for multiple hypothesis testing.

Stepwise forward logistic regressions and ROC curves were used to examine biomarkers as classifiers of sex and reproductive aging status, and to calculate associated relative risk (RR) and 95% confidence intervals (C.I.) at p<0.05.

## Results

### Participants

Of the 78 participants aged 40–60 available for analysis, we excluded 9 participants who did not complete follow-up scans or reproductive aging records. Ten additional women were excluded due to medical reasons including ovariectomy/hysterectomy (3), thyroid disease (3), history of cancer (1), and HRT (3).

The remaining 59 participants were examined. These included 41 women (15 PRE, 14 PERI, and 12 MENO) and 18 men. Two of the baseline PRE women transitioned to PERI, and 2 PERI women transitioned to MENO during the study.

Participants’ characteristics are found in **Table 1**. As expected, the MENO group was older than PERI and PRE (p < .001). There were no other group differences for clinical and demographical measures, APOE4 genotype frequency, and presence of family history of AD. The MENO group showed a trend towards a higher frequency of subjective memory complaints compared to PRE women and men (p ≤ .10).

**Table 1:** Baseline demographic and clinical characteristics by group.

	Women			Men
	PRE	PERI	MENO	
N	15	14	12	18
Age, years range	47(5) 40–55	53(4) 45–60	58(2)^b^ 55–60	52(6) 42–60
Education, years	16(2)	16(2)	17(2)	17(2)
Family history of AD, No. (%) positive	11 (73%)	11 (79%)	10 (83%)	14 (78%)
*APOE*4 status, No. (%) positive	7 (47%)	7 (50%)	5 (42%)	9 (50%)
Ethnicity, No. (%) White	11 (73%)	10 (75%)	10 (83%)	15 (83%)
Subjective complaints, No. (%) positive	9 (60%)	12 (86%)	11 (92%)	12 (67%)
Global Cognition (Z scores)	-.02(.50)	-.01(.42)	-.14(.46)	-.01(.56)
Time to follow-up, years range	3(.5) 2–3.5	3(.5) 2–3.2	2(.4) 2–3.3	3(.5) 2–3.5
Hypertension, No. (%) positive	1 (7%)	1 (7%)	3 (25%)	4 (22%)
Body Mass Index	25(5)	25(4)	23(7)	27(4)
Hip to waist ratio (unitless)	1.23(.11)	1.25(.12)	1.24(.14)	1.09(.05) ^a^
QUICKI scores (unitless)	.17(.02)	.18(.02)	.17(.02)	.15(.01) ^b^^,^^c^
Cholesterol/HDL (unitless)	3.26(.58)	3.22(.64)	3.10(.91)	4.03(.96) ^a^

Values are means (SD) unless otherwise specified.

^a^ Different from all female groups

^b^ Different from PRE

^c^ Different from PERI, p<0.05

Abbreviations: MENO = postmenopausal women, PERI = perimenopausal women, PRE = premenopausal women, QUICKI = Quantitative insulin-sensitivity check index

Men had higher waist-to-hip measurements and plasma cholesterol/HDL compared to all female groups (p < .04), and lower QUICKI scores (e.g., higher insulin resistance) compared to PRE and PERI women (p < .05).

### Effects of menopause on cognition

None of the clinical and vascular risk measures were associated with changes in global cognition and female-specific outcomes.

Accounting for age and APOE status, there were no group differences in global cognition at baseline or over time (**Table 2**). After including vascular risk factors, men exhibited higher rates of decline in global cognition as compared to the MENO group (p < .01).

**Table 2:** Adjusted means of cognitive and biomarker change by group.

		Model 1		Model 2	
		Adjusted mean	SD	Adjusted mean	SD
**Cognitive scores**					
Global cognition	PRE	-.164	.038	-.050	.034
	PERI	.092	.020	.031	.059
	MENO	.009	.029	.015	.016
	Men	-.012	.050	-.102^**d**^	.033
Estrogen-dependent memory	PRE	-.114	.062	-.056	.053
	PERI	-.159^**c**^	.038	-.053	.039
	MENO	-.213^**c**^	.074	-.050	.096
	Men	.097	.123	.079	.138
Estrogen-dependent higher-order processing	PRE	.030	.122	.116	.123
	PERI	.776	.267	.358	.254
	MENO	-.647 ^**a**^^,^^**b**^^,^^**c**^	.107	-.195 ^**a**^^,^^**b**^^,^^**c**^	.107
	Men	.097	.153	.059	.159
**AD biomarkers**					
MRI hippocampal volume	PRE	58.64	10.11	36.77	10.81
	PERI	-38.76 ^**a**^	35.69	16.90	15.55
	MENO	-71.36 ^**a**^^,^^**c**^	18.99	-99.73 ^**a**^^,^^**b**^^,^^**c**^	16.73
	Men	20.10	19.16	32.39	8.99
FDG posterior cingulate SUVR	PRE	.009	.004	.015	.003
	PERI	-.002	.005	.0006	.005
	MENO	-.001	.005	.002	.011
	Men	.003	.005	.009	.006
FDG frontal SUVR	PRE	-.009	.010	-.005 ^**c**^	.007
	PERI	-.017 ^**c**^	.007	-.008 ^**c**^	.004
	MENO	-.037^**a**^^,^^**b**^^,^^**c**^	.005	-.036^**a**^^,^^**b**^^,^^**c**^	.008
	Men	-.002	.002	.005	.004
PiB posterior cingulate SUVR	PRE	-.048	.022	.007	.017
	PERI	.024	.033	.003	.028
	MENO	.065 ^**a**^	.021	.055 ^**a**^	.052
	Men	.021	.027	.080 ^**a**^	.030
PiB frontal SUVR	PRE	.002	.007	-.039	.009
	PERI	.048^**a**^^,^ ^**c**^	.009	.031^**a**^	.010
	MENO	.021^**a**^^,^ ^**c**^	.015	.064^**a**^	.031
	Men	-.003	.021	-.012	.018

Abbreviations: see legend to Table 1. Adjusted means reflect the mean cognitive and biomarker change in each measure adjusted for possible confounds, as computed with TMLE (see Appendix).

^a^ Different from PRE

^b^ Different from PERI

^c^ Different from Men

^d^ Different from MENO, p<0.05 corrected for multiple comparisons

Model 1: outcome variables are adjusted for age and APOE status.

Model 2: outcome variables are adjusted for age, APOE status, and vascular risk measures. Cognitive variables are further adjusted for AD biomarkers.

In contrast, estrogen-dependent tests analysis indicated that the MENO and PERI groups had higher rates of memory decline compared to males (p < .02). The MENO group also exhibited higher rates of decline in higher-order processing compared to all other groups (p < .04; **Table 2**).

### Effects of menopause on amyloid deposition

At baseline, older age was associated with higher amyloid levels in PCC and frontal cortex across all subjects (p < .04). Longitudinally, APOE4 status was associated with higher rates of amyloid deposition in frontal regions (p = .02). Education and vascular measures were not associated with amyloid deposition.

Adjusting for confounders, the MENO group exhibited higher rates of amyloid deposition in both PCC and frontal cortex compared to the PRE and male groups (p ≤ .001; **Table 2**). The PERI group exhibited higher rates of amyloid deposition in frontal cortex compared to the PRE group and men (p < .011), whereas men showed higher rates of amyloid deposition in PCC compared to the PRE group (p < .02; **Table 2**).

Over the 3 years, frontal PiB uptake increased by an average of 6.3% in the MENO group, and 4.5% in the PERI group. In contrast, frontal PiB uptake did not show significant increases in the PRE and male groups (<1% change). PCC PiB uptake increased by an average of 8.6% in the MENO group and by 6.2% in the male group, and showed no significant changes in the PRE and PERI groups (<1.3% change).

### Effects of menopause on CMRglc

Older age was negatively associated with baseline frontal CMRglc (p < .04), but was not associated with baseline PCC CMRglc or CMRglc change. No other clinical and vascular measures were associated with CMRglc.

Correcting for age, the MENO group exhibited higher rates of CMRglc decline in frontal cortex compared to all other groups (p < .009; **Table 2**). The PERI group also showed higher rates of frontal CMRglc decline than men (p < .009).

Longitudinally, frontal CMRglc declined by an average of .017 SUVR per year (SD = .006) in the PERI group and by .037 SUVR per year (SD = .005) in the MENO group, corresponding to an average decrease from baseline of 4.0% and 6.1% respectively. CMRglc did not decline over the 3-year period in PRE women nor in males (<1% change).

### Effects of menopause on hippocampal volume

As expected, larger intracranial volume was a predictor of larger hippocampal volume (p < .001). Older age showed marginal associations with hippocampal volume at baseline and over time (p < .09). Education, APOE4 status and vascular measures were not associated with hippocampal volumes.

In both models, the MENO group exhibited the highest rate of hippocampal volume loss compared to all groups (p’s < .001; **Table 2)**. Hippocampal volumes decreased by an average of 3.3% in the MENO group, and showed minimal to no decline in the other groups (<1% change).

### Assessment of overall AD-burden across groups

**Fig 1:**
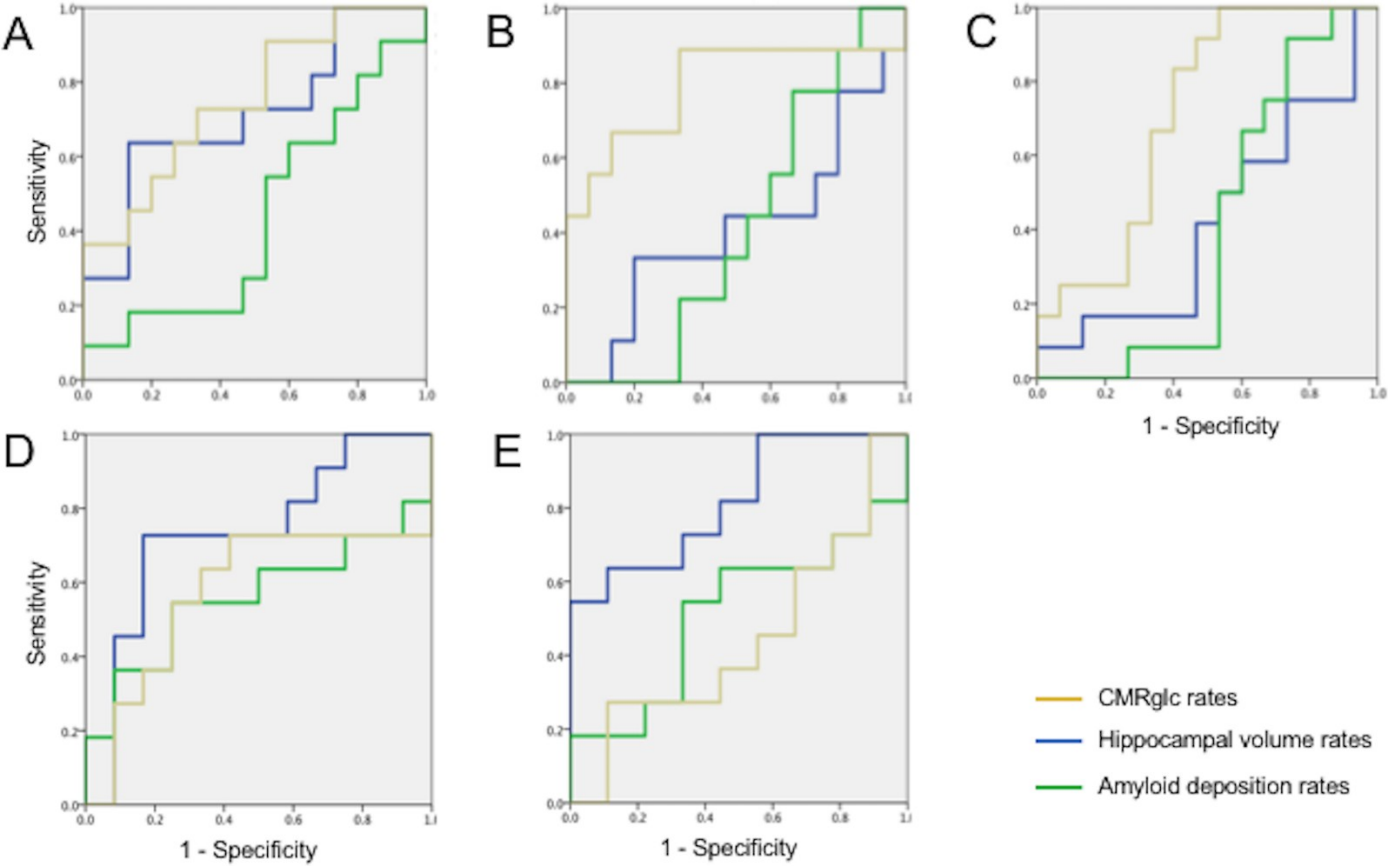
ROC curves showing group separation as predicted by AD biomarkers. A) MENO vs. males; B) PERI vs. males; C) PRE vs Men; D) MENO vs PERI; E) MENO vs PRE. Graphs show group separation as predicted by 3-year changes in FDG-PET CMRglc in frontal cortex (blue; age-adjusted measures), PiB-PET amyloid load in frontal cortex (orange; age-adjusted measures), and hippocampal volume (green; age and TIV-adjusted measures).

Logistic regression results are summarized in **Fig 1**.

*MENO vs Men*. Stepwise forward analysis identified frontal CMRglc rates as the most accurate predictors of MENO status vs. men (p = .010; **Fig 1A**). Adding hippocampal volume rates increased the overall accuracy (p_increment_ < .001). The combination of frontal CMRglc and hippocampal volume classified MENO vs. men with 75% sensitivity and 100% specificity, for a total accuracy of 90% (p = .003; relative risk, RR = 27.8, 95% C.I. 1.77–436.5).

*PERI vs Men*. Frontal CMRglc rates were the only predictors of PERI vs. men, yielding 75% accuracy (*X*^2^_(1)_ = 5.98, p = .014; RR = 3.20, 95% C.I. 1.03–9.97; **Fig 1B**).

*PRE vs Men*. Frontal CMRglc rates were borderline predictors of PRE vs. men, yielding 50% accuracy (p = .067; RR = 1.02, 95% C.I. 0.56–1.89; **Fig 1C**).

*MENO vs PERI*. Hippocampal volume rates were the only predictors of MENO vs. PERI with 73% accuracy (*X*^2^_(1)_ = 4.29, p = 0.038; RR = 3.11, 95% C.I. 1.06–9.16; **Fig 1D**).

*MENO vs PRE*. Hippocampal volume rates were the only predictors of MENO vs. PRE with 78% accuracy (*X*^2^_(1)_ = 6.41, p = 0.011; RR = 5.00, 95% C.I. 1.30–19.3; **Fig 1E**).

## Discussion

In our earlier cross-sectional analyses[13],[14], multi-modality brain imaging detected emergence of a female-specific AD-endophenotype characterized by decreased brain glucose bioenergetics, increased Aβ deposition, and gray volume loss in PERI and MENO women. We now find that AD biomarker abnormalities progressed in PERI and MENO women during the 3-year interval between baseline and follow-up scans. Specifically, the MENO group showed increasing abnormalities in all modalities, and the PERI group showed increasing Aβ deposition and hypometabolism in frontal cortex.

**Fig 2:**
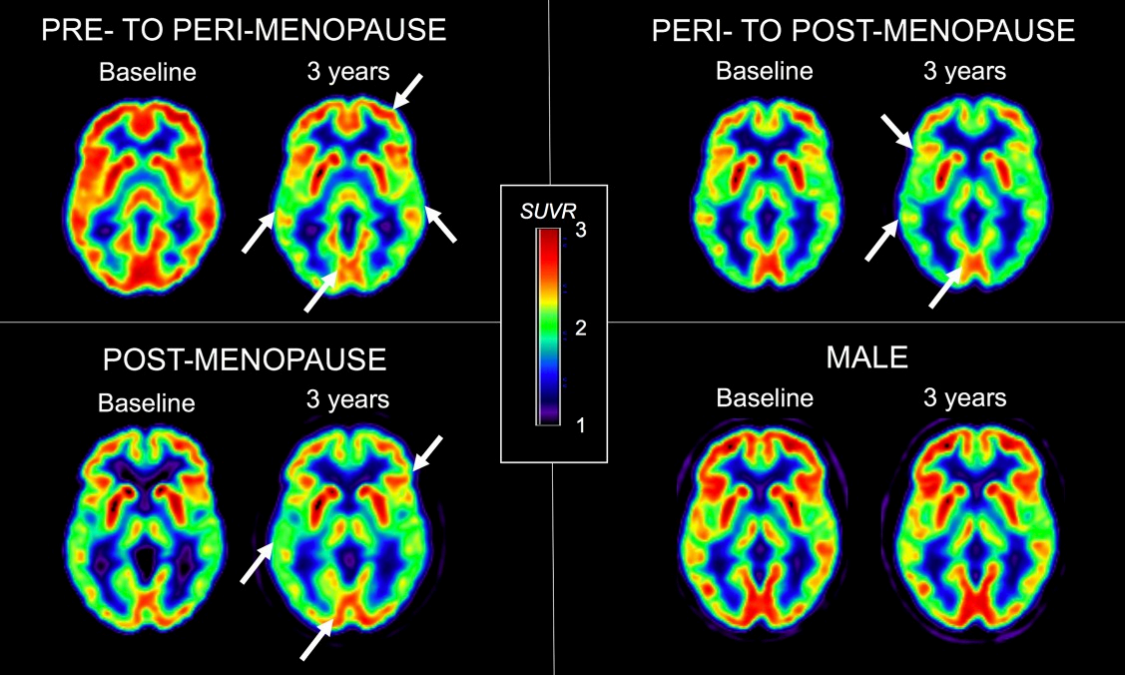
Baseline and follow-up FDG-PET scans in 4 representative cases. Top left: A 52 year-old woman (14 years education, APOE 3/4 carrier, CDR 0, MMSE 30) who was premenopausal at baseline and became perimenopausal by the follow-up exam. Top right: A 43 year-old woman (16 years education, APOE 3/3 carrier, CDR 0, MMSE 29) who was perimenopausal at baseline and became postmenopausal by the follow-up exam. Bottom left: A 59 year-old woman (16 years education, APOE 3/3 carrier, CDR 0, MMSE 30) who was postmenopausal at baseline. Bottom right: A 55 year-old man (14 years education, APOE 3/4 carrier, CDR 0, MMSE 30). Arrows point to areas of progressive CMRglc reductions in the follow-up vs. baseline scans. FDG measures are standardized uptake volume ratios (SUVR) adjusted for global uptake.

Biomarker changes preceded any evidence of clinical AD symptoms, and were independent of other AD risk factors such as age, APOE4 genotype, and vascular comorbidity. These longitudinal findings suggest that: 1) neurodegenerative changes may arise at an earlier age in women than in men; namely, during the PTMT; 2) Aβ deposition affects middle-aged women and men, though it was restricted to PCC in men and affecting both PCC and frontal cortex in MENO women, suggesting more widespread distribution in the latter group; 3) the identified female-specific AD endophenotype is one of progressive metabolic decline in excess of Aβ deposition and hippocampal atrophy; and 4) the frontal cortex, a brain region known to be metabolically vulnerable to aging and AD[20], is preferentially affected in women, suggesting that the PTMT may act as an accelerator of brain aging. This effect is exemplified in **Fig 2**.

These data indicate that female endocrine transitions influence brain bioenergetics and are in agreement with mechanistic preclinical pathway evidence of a bioenergetic crisis in the female brain that emerges specifically during the PTMT[4,25–28]. In animal studies, estrogenic control of CMRglc is disassembled during the PTMT, leading to an adaptive activation of ketone body metabolism to generate ATP[26]. Over time, continued reliance on ketone bodies can lead to compromised mitochondrial energetic function, white matter catabolism[27], and cellular apoptosis[25].

Given the known relationships between brain AD biomarker abnormalities and onset of AD clinical symptoms[29], our data indicate that disruption of brain bioenergetics during the PTMT may, in part, account for the increased AD risk in women. Chronologically, age of menopause maps onto the time course for initiation of the prodromal phase of AD, which typically begins 15–20 years before clinical diagnosis[30]. Menopausal changes therefore coincide with the timespan between average age of menopause (mid-50s) and average age of AD diagnosis (mid-70s).

We also observed differential effects of sex and endocrine status on regional Aβ deposition. Although both the MENO and male groups exhibited increasing PiB retention in PCC, only the MENO group had a concurrent PiB increase in frontal cortex. Additionally, the PERI group exhibited higher rates of frontal Aβ deposition than males, further implicating the frontal cortex as particularly vulnerable to endocrine aging and risk of AD progression in women. Braak’s postmortem staging[31] indicated that the frontal cortex is affected by amyloid deposition earlier than PCC, which suggests that MENO women may be at a more advanced stage of brain amyloidosis than men of similar age. More studies using multiple ROIs or whole-brain voxel-based analysis (VBA) are warranted to assess whether Aβ deposition is indeed more widespread in MENO women than men, as also suggested by our previous cross-sectional work using VBA [13].

In keeping with previous studies with similar middle-aged cohorts [32,33], PiB measures were examined as continuous rather than dichotomous variables. Using this method, we observed quantitative differences across sex and female groups, which included so-called “PiB-negative” patients. These findings are consistent with previous evidence that the PiB signal is sensitive to detect emerging, albeit mild Aβ accumulation within middle-aged people at risk for AD [17].

Men showed somewhat higher rates of global cognitive decline than women, in keeping with previous observations that women typically outperform men for several years until postmenopause[6]. However, our findings show that the MENO group exhibited higher rates of decline on select estrogen-dependent memory and higher-order processing tests as compared to males, indicating that these measures may be better suited to capture early cognitive declines in women. Evidence that MENO and to a lesser extent PERI groups exhibited progression of AD biomarkers in the absence of severe cognitive declines is consistent with other studies showing lack of overall associations between imaging biomarkers and neuropsychological in asymptomatic at-risk individuals[32,34,35]. Further, the fact that our patients were all cognitively normal and high-school graduates could have resulted in a “ceiling-effect”. We chose to enroll participants with ≥12 years education to ensure a relatively homogeneous socioeconomic status, and as such minimize performance variability associated with low education. Studies of community-based studies with higher socio-economic and educational variability are warranted to determine whether biomarker abnormalities observed in perimenopausal and postmenopausal women are predictive of future cognitive decline.

Several questions remain to be answered. First, it remains to be established whether neurodegenerative changes arise downstream of ongoing Aß dysmetabolism in women, in keeping with hypothetical models of AD[29], or whether Aß deposition is exacerbated as a consequence of a PTMT-related bioenergetic crisis[4,25–28].

Our determination of menopausal state in the absence of hormonal confirmation is vulnerable to error. Our classification was based on self-report, clinical judgment, and established diagnostic criteria known to be in agreement with clinical and lab findings[16], which reduce potential for misclassification. While we consider it likely that changes in menstrual cycle frequency reported by our participants reflect actual menopausal status, our PERI group may have included more women already undergoing menopause. Likewise, our PRE women may have been undergoing perimenopause. This would, however, conservatively reduce power in detecting differences between groups. Our findings of more pronounced biomarker abnormalities in MENO, and intermediate changes in PERI women are consistent with preclinical data[6,36],[37], providing support that our group assignment were likely correct.

None of the women included were on HRT. Clinical trials have shown that HRT is effective at preserving CMRglc in AD-regions, especially if initiated prior to menopause[38,39], whereas HRT can be deleterious if initiated after menopause[40,41] or in type 2 diabetic women[38,42,43]. Our results support further investigation of the potential efficacy of estrogen-based therapies in mitigating bioenergetic declines in women.

Due to the relatively small sample size, we were unable to test for interactions between groups and APOE4 genotype, a well-established genetic risk factor late-onset AD known to increase AD risk[1,44], brain atrophy [45] and Aß deposition [13] much more strongly in women than in men. More work is needed to specifically examine the impact of APOE4 on AD biomarker progression during the menopause transition[46].

Our results are pertinent to healthy, middle-aged participants without severe cerebrovascular or metabolic disease. Studies with larger samples and longer follow-ups are necessary to assess the generalizability of these findings in community-based populations with higher socio-economic and medical status variability.

Overall, these findings provide a plausible rationale for greater prevalence of AD in women due to earlier initiation of pathology during the aging process, and indicate a time frame of the PTMT for early intervention to prevent and delay progression of AD in women.
